# Regional wall function before and after acute myocardial infarction; an experimental study in pigs

**DOI:** 10.1186/1532-429X-16-S1-P189

**Published:** 2014-01-16

**Authors:** Ulrika S Pahlm, Joey F Ubachs, Einar Heiberg, Henrik Engblom, David Erlinge, Matthias Götberg, Håkan Arheden

**Affiliations:** 1 Department of Clinical Physiology, Lund University, Lund, Sweden; 2 Centre for Mathematical Sciences, Lund University, Lund, Sweden; 3 Department of Cardiology, Lund University, Lund, Sweden

## Background

Acute myocardial infarction (AMI) is a major cause of death worldwide and affects both global and regional left ventricular function. Regional function can be determined by CMR i.e. wall thickening, and velocity encoded (VE) strain analysis. Aim: The aims of this study were to investigate how regional myocardial wall function, assessed by CMR velocity encoded strain and regional wall thickening, change after acute myocardial infarction and to determine if these variables can be used to differentiate between ischemic, adjacent and remote myocardium after experimentally induced myocardial infarction.

## Methods

Ten pigs underwent baseline CMR study for assessment of wall thickening and VE-strain. Ischemia was then induced for 40-minutes by intracoronary balloon inflation in the left anterior descending coronary artery. During occlusion, 99mTc tetrofosmin was administered intravenously and myocardial perfusion SPECT (MPS) was performed for determination of the ischemic area, followed by a second CMR study. Based on ischemia seen on SPECT, the 17 AHA segments of the left ventricle was divided into 3 different categories (ischemic, adjacent and remote). Regional wall function measured by wall thickening and VE strain analysis was determined before and after ischemia.

## Results

**Figure 1:**
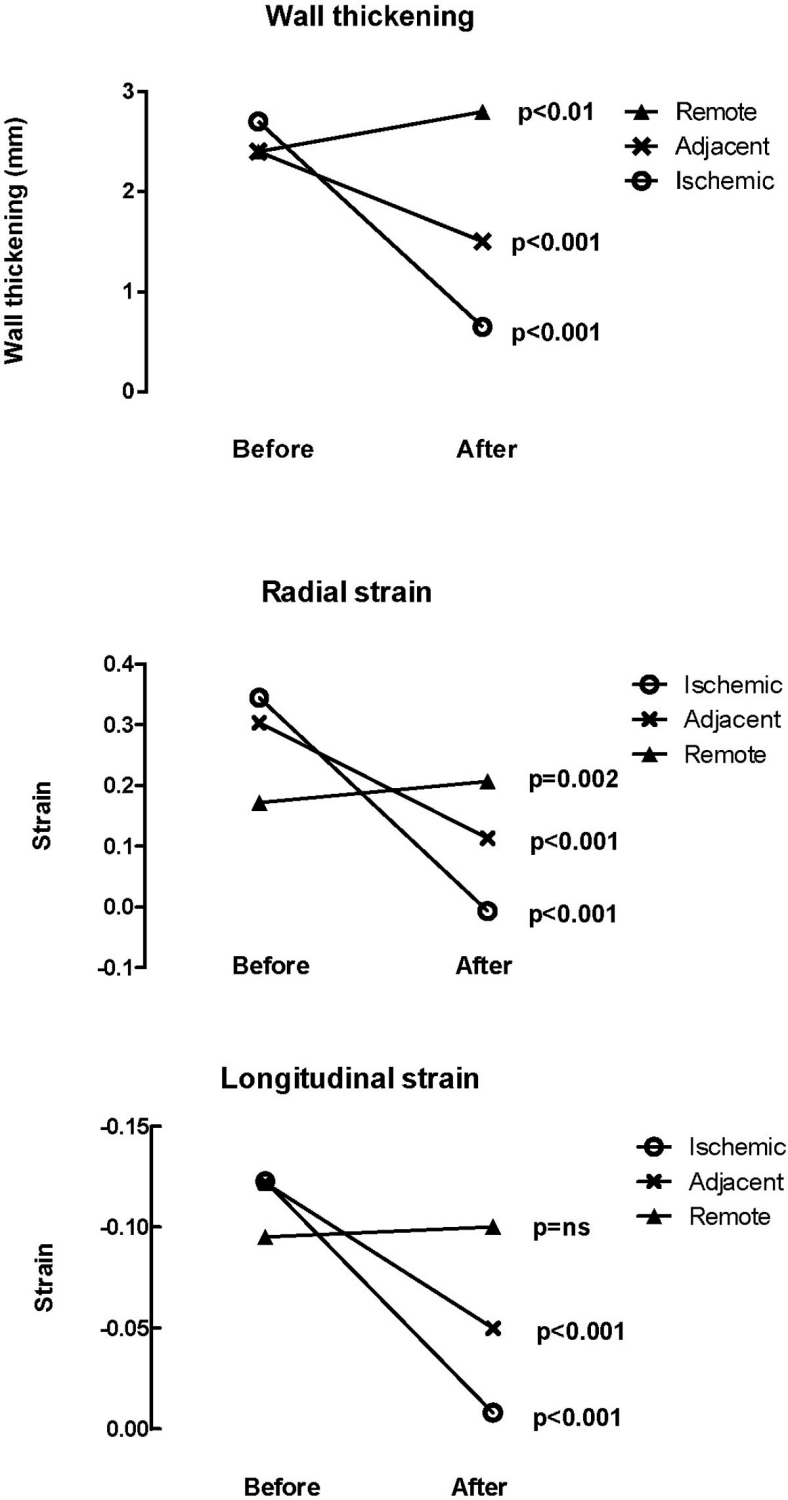
**Changes in wall thickening (top row), radial strain (middle row), and longitudinal strain (bottom row) before and after ischemia**.

**Figure 2:**
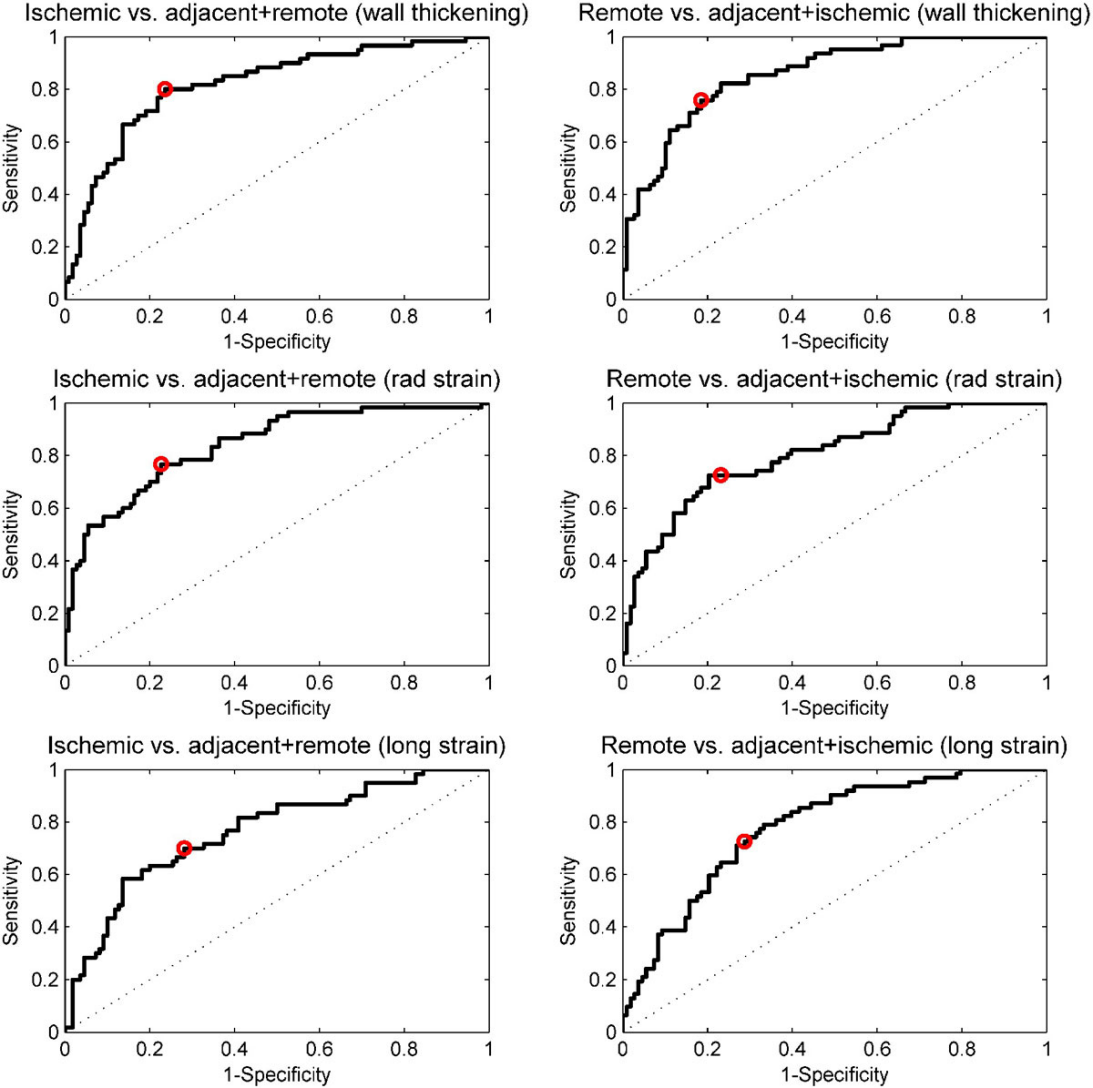
**ROC analysis determining threshold**. Rows show results for wall thickening, radial strain and longitudinal strain, respectively. Left column shows differentiation between ischemic and non-ischemic areas. Right column shows differentiation between remote and non-remote areas. Marker shows sensitivity and specificity for optimal thresholds.

Changes in wall thickening and strain before and after ischemia are shown in Figure 1. Mean wall thickening decreased significantly in the ischemic (from 2.7 mm before ischemia to 0.65 mm, p < 0.001) and adjacent segments (from 2.4 to 1.5 mm p < 0.001). In remote myocardium, wall thickening increased significantly (from 2.4 mm to 2.8 mm, p < 0.01). In ischemic and adjacent areas, both radial and longitudinal strain was significantly decreased after ischemia (p < 0.001). ROC analysis was performed to determine thresholds to distinguish between the different regions. Sensitivity for determining ischemic regions ranged from 70-80%, and specificity from 72%-77% (Figure 2). There was a 9% increase in left ventricular mass after ischemia primarily to swelling of the ischemic myocardium.

## Conclusions

Both wall thickening and VE strain decrease significantly in both the ischemic and adjacent myocardium acutely after reperfused coronary occlusion. Differentiation thresholds for ischemic, adjacent and remote myocardium, could be determined. Thresholds will however, have limited applicability due to low sensitivity and specificity.

## Funding

Swedish Research Council, Region of Scania, Swedish Heart-Lung Foundation and the Medical Faculty at Lund University.

